# Who should talk with patients about their end-of-life care wishes? A nationwide survey of the Hungarian population

**DOI:** 10.1080/02813432.2022.2057055

**Published:** 2022-04-04

**Authors:** Csilla Busa, Eva Pozsgai, Judit Zeller, Agnes Csikos

**Affiliations:** a Institute of Primary Health Care, Department of Palliative Care, University of Pecs Medical School, Pecs, Hungary; b Doctoral School of Health Sciences, University of Pecs Faculty of Health Sciences, Pecs, Hungary; c Faculty of Law, University of Pecs, Pecs, Hungary

**Keywords:** End-of-life conversation, end-of-life care wishes, advance care planning, general population, general practitioners, family members, communication needs and opportunities

## Abstract

**Objective:**

To explore the needs and opportunities of the general population to communicate their end-of-life care wishes and to investigate what roles are assigned to healthcare providers and family members in end-of-life care discussions.

**Design:**

A cross-sectional social survey was carried out in Hungary. Descriptive analysis and analysis of variance were performed.

**Setting:**

Nationwide survey of the Hungarian general population.

**Subject:**

The sample (*n* = 1100) was designed to represent the adult population as per distribution by gender, age and geographical region.

**Main outcome measures:**

Needs and opportunities of the general population to communicate end-of-life care wishes.

**Results:**

72% of participants found it important to discuss their end-of-life care wishes with someone. Six out of ten believed that it was also the GPs’ task to talk with the patients about their end-of-life care wishes. An almost equal level of engagement was expected from healthcare providers (80%) -especially physicians (72%)- and family members (75%) in end-of-life conversations. However, only 36% of participants felt that there was someone among their healthcare providers, and 56% of them had a family member or friend with whom they could speak openly about death, dying and preparing for death.

**Conclusion:**

Compared to their needs, the general population had fewer opportunities to speak about death, dying and preparing for death. Training programs for healthcare providers, particularly GPs, and public awareness campaigns may support the broader application of advance care planning in Hungary.

**Key Points**
- Current guidelines recommend that GPs initiate advance care planning discussions. However, little is known with whom the general population wish to discuss their end-of-life care preferences and with whom there is an opportunity to do so.
- An almost equal level of engagement was expected from healthcare providers -especially physicians- and family members in end-of-life conversations. Most of the general population thought that participation in end-of-life discussions was also the GPs’ task.
- The majority of participants reported that there was no one among their healthcare providers and a sizable minority felt that there was no one among their family members or friends with whom they could talk openly about death, dying, and preparing for death.
- The highest levels of unmet needs regarding end-of-life conversations with healthcare providers were found among those who considered it important to discuss their end-of-life care wishes.

## Introduction

Since chronic conditions have become the leading causes of death, people can expect to endure longer periods of terminal illness, which provides patients an opportunity to prepare for the last period of their lives. Earlier studies have revealed that many people had concerns and wishes regarding care at the end of life and would like to participate actively in decision-making [1,2]. The general population’s views on preferences regarding end-of-life (EOL) care are particularly significant since these people might be involved in decision-making later on as patients or as relatives [1,3,4]. The Council of Europe has also recommended that member states encourage their citizens’ self-determination about their future care [5]. However, most people do not share their wishes with their loved ones or healthcare providers (HCPs) [1,2,6]. Cultural norms, religious beliefs, characteristics of the communication in the healthcare system and the families, and personal fears might be the reasons for avoiding death-related topics [7].

EOL conversations generally indicate discussions about patients’ wishes regarding future care. Advance care planning (ACP) is a formalized process of EOL conversations about the individual’s values and care preferences with his or her family members and HCPs [8]. ACP improves communication, enhances interdisciplinary teamwork, increases the quality of life, and the concordance between patients’ preferences and the delivered care [9]. Several studies have confirmed that people wished physicians would start these conversations [2,7]. Current guidelines recommend that general practitioners (GPs) initiate ACP discussions [10,11]. GPs encounter a large number of patients for whom ACP would be beneficial [10,12], they are aware of the patients’ medical history and family context, and they may bring up this topic at the right time in a non-threatening manner [13]. Despite the benefits, the level of implementation of ACP in primary care has remained low [14]. Insufficient time, limited knowledge and confidence, fear of a discussion potentially destroying hope, unclear roles [14,15] and suboptimal collaboration between GPs and other specialists are the most common barriers [16].

GPs in Hungary have the overall responsibility to provide home care for dying patients in collaboration with attending physicians and palliative-hospice care specialists. Two-thirds of GPs provide this type of care sometimes, while one-third always does so [17]. A pilot survey pointed out that Hungarian GPs felt responsible for exploring patients’ needs regarding EOL care [18]. More than four-fifths of the surveyed GPs thought it was a GP’s task, three-quarters of them believed the attending physician should do so, and nearly nine out of ten thought that family members should talk with patients about this topic [18]. The pilot survey has also revealed that most Hungarian GPs felt unprepared to talk about EOL issues due to their limited -theoretical and practical- knowledge and skills [18]. People in Hungary have a permanent GP with whom they have a longstanding relationship. Under universal health coverage, GP service, specialist service and palliative care are available for patients without any out-of-pocket cost. In line with international recommendations, Hungarian GPs would be in an ideal position to conduct ACP discussions. Notwithstanding, ACP is extremely underused in primary care; EOL conversation has no formalized practice in the Hungarian healthcare system, and patients’ EOL care preferences are not systematically assessed [19].

The level of awareness regarding EOL planning in Hungarian society is low, and the term ‘advance care planning’ is unknown to the general public [19]. Nearly two-thirds of Hungarian adults wished to die at home, and most of them thought that there would be someone among their family members who could provide care for them [20]. In fact, two-thirds of the Hungarian population die in a hospital [21], and the majority of them presumably do not communicate their EOL care wishes [19,22]. Communication on EOL issues is still an under-researched topic in Hungary. Until now, no comprehensive study has been conducted investigating the views and needs of the general public regarding EOL conversations. The present study aimed to explore the needs and opportunities of the Hungarian general population to communicate their EOL care wishes and to investigate what roles are assigned to physicians (including GPs) and family members in EOL care discussions. A cross-sectional social survey was performed on a representative sample of the general population in Hungary.

## Methods

### Questionnaire

The questionnaire was developed by a multidisciplinary expert panel and was pilot-tested. The wording of the questions was kept plain and clear. As the term ‘advance care planning’ is not known to the Hungarian general population, descriptive terms for EOL conversation (e.g. ’to discuss EOL care wishes in advance’, ’to talk about EOL care wishes’) were used instead. The final version consisted of 31 items: dichotomous and multiple-choice questions, and questions with Likert scale answers.

### Sampling method and data collection

The target population in this study was the Hungarian adult population. A stratified sampling technique was applied to design a sample of 1100 participants representing the total Hungarian adult population concerning distribution by gender, age and geographical region. The primary sampling units were geographic regions, and the secondary sampling units were the households selected by the random route method. Inclusion criteria for participation were age 18 or above, residence in Hungary, fluency in Hungarian and consent to participate. Questionnaires were completed face-to-face by professional interviewers at the participants’ homes. Data collection ended when the predefined sample size was achieved.

### Consent

The study was carried out according to Hungarian legislation on privacy. The participants were informed about the objectives of the research. Anonymity was insured and participation was voluntary. Informed consent were electronically recorded by the interviewers.

### Statistical analysis

Post-stratification weighting process was performed, the sample was balanced to match the adult population parameters using data from the Hungarian Population Census. Statistical analysis was undertaken using IBM SPSS Statistics for Windows (version 24.0) software. Descriptive analysis and analysis of variance were performed. Percentages for categorical variables, means, standard deviations and confidence intervals for continuous variables were used to describe the results. Statistical significance was assessed by using Pearson Chi-Square Test and Fisher's Exact Test for the categorical variables and ANOVA test for the continuous variables. *P*-value <0.05 was considered statistically significant.

## Results

### Sociodemographic characteristics of the participants

The male to female ratio was 47% to 53%. Participants over 55 years constituted the largest group in the sample (38%), and the mean age was 47.51 years. More than half (53%) of participants had completed secondary school. The mean score of self-rated health (SRH) was 7.85 (Table 1).

**Table 1.** Selected sociodemographic characteristics of the participants.

Sociodemographic characteristics	Participants *n* (%)
Gender	
Male	513 (47)
Female	587 (53)
Age group	
18–34 years	303 (28)
35–54 years	377 (34)
55+ years	420 (38)
Mean age in years	47.51
[95% CI]	[46.50–48.53]
Std. Dev.	17.17
Highest attained level of education	
Primary	346 (31)
Secondary	584 (53)
College or university	169 (15)
No answer	1 (0)
Self-rated health (1: poor … 10: excellent)	
1–6	172 (16)
7	127 (12)
8	218 (20)
9	169 (15)
10	187 (17)
No answer	227 (21)
Mean score	7.85
[95% CI]	[7.73–7.97]
Std. Dev.	1.86

CI: Confidence Interval.

### Importance of EOL care discussions

More than seven out of ten participants found it important to discuss their EOL care wishes with someone in advance, while they were still in good health. Nearly one-quarter of these (24%) regarded it as ‘very important’ to talk to someone about their choices (Table 2). A third (34%) of participants with higher education considered EOL conversations important, while this proportion among participants with secondary or primary education was lower (22% and 23%). Among those participants who found a discussion on EOL care ‘very important’, the mean age (46.97 years) and the mean SRH score (7.62) were also significantly lower than the sample means (47.51 years; SHR 7.85). Differences by gender, marital status and composition of the household were not statistically significant.

**Table 2.** Importance of EOL care discussion.

Sociodemographic characteristics of the participants	Do you find it important to discuss your EOL care wishes with someone in advance, while you are still in good health?					
	Very important	Important	Not important	Not important at all	DNK	*p*-value
	264 (24)	524 (48)	145 (13)	100 (9)	67 (6)	–
	Important 788 (72)		Not important/DNK 312 (28)			
Highest attained level of education						–
Primary	78 (23)	164 (48)	38 (11)	46 (13)	19 (6)	*<*0.001^a^
Secondary	128 (22)	285 (49)	84 (14)	46 (8)	41 (7)	
College or university	57 (34)	75 (44)	23 (14)	8 (5)	7 (4)	
Mean age in years	46.97	48.61	49.40	43.22	43.47	0.007^b^
[95% CI]	[45.11–48.83]	[47.07–50.15]	[46.61–52.20]	[40.04–46.40]	[39.02–47.92]	
Std. Dev.	15.35	17.95	17.05	16.08	18.27	
Mean score of the self-rated health	7.62	7.79	8.03	8.17	8.37	0.018^b^
[95% CI]	[7.36–7.89]	[7.61–7.96]	[7.71–8.35]	[7.76–8.59]	[7.86–8.88]	
Std. Dev.	1.99	1.78	1.65	1.98	1.91	

DNK: Do not know.

CI: Confidence Interval.

^a^ Pearson Chi-Square Test.

^b^ ANOVA Test.

### Whose task is it to talk with patients about their EOL care wishes?

Three-quarters of participants (75%) believed that it was the family members’ task to talk with the patient about his or her EOL care wishes (Table 3). Two-thirds of participants (66%) thought that the attending physician, 58% that the GP, and 56% that a trained facilitator should talk with the patient about this topic. (Multiple choices were allowed.) Overall, 72% of participants answered ‘physicians’ and 80% mentioned ‘HCPs’ as carers whose task it was to talk with the patients about their choices.

**Table 3.** Needs (tasks) regarding EOL conversations.

Whose task is it to talk with patients about their EOL care wishes? (You may choose more than one answer.) ‘Yes’ answers; *n* (%*)			Importance of EOL care discussion *n* (%)		
			Important	Not important/DNK	*p*-value
Family members		829 (75)	663 (84)	166 (53)	*<*0.001^a^
Healthcare providers	Attending physician	720 (66)	559 (71)	161 (51)	*<*0.001^a^
	General practitioner	638 (58)	515 (65)	123 (40)	*<*0.001^a^
	Trained facilitator	613 (56)	483 (61)	130 (42)	*<*0.001^a^
Mean number of carers mentioned		2.54	2.82	1.86	*<*0.001^b^
[95% CI]		[2.47–2.62]	[2.73–2.90]	[1.70–2.01]	
Std. Dev.		1.35	1.23	1.40	
Physician(s) (Attending physician and/or General practitioner)		794 (72)	618 (79)	176 (57)	*<*0.001^a^
Healthcare providers (One or more healthcare providers)		876 (80)	676 (86)	200 (64)	*<*0.001^a^
Both family members and healthcare providers (Family members and one or more healthcare providers)		710 (65)	586 (74)	124 (40)	*<*0.001^c^

*The percentages were calculated in proportion to the total number of participants (*n* = 1100). Multiple choices were allowed therefore the sum of the answers is not matching the number of participants.

DNK: Do not know.

CI: Confidence Interval.

^a^Fisher's Exact Test.

^b^ANOVA Test.

^c^Pearson Chi-Square Test.

According to participants, EOL conversations require a collaboration between the family and the HCPs. The mean number of carers mentioned who should participate in EOL discussions was 2.54. Two-thirds (65%) of participants believed that both the family members’ and the HCPs’ task was to talk with the patients regarding their EOL care wishes.

In the participants who found EOL conversations important, family members and HCPs were cited in very similar proportions (84% vs 86%). Three-quarters (74%) of participants thought that both family members and HCPs should participate in EOL discussions. In this group, the proportion with the response ’GP’ was also higher than within the total sample (65% vs 58%).

### The difference between people’s needs and opportunities for EOL discussions

More than half (56%) of participants reported that there was someone in their family or friends with whom they could speak openly about death, dying and preparing for death. Slightly more than one-third (36%) had such a person among their HCPs. Three out of ten participants believed that they had someone in their family or friends and among their HCPs with whom they could freely discuss this topic. The participants who found EOL conversations important represented higher percentages than those who did not regard it as important or answered ‘I do not know’ (Table 4).

**Table 4.** Opportunities to talk about EOL care.

Is there anyone in your family or friends / among your health care providers with whom you could speak openly about death, dying and preparing for death? ‘Yes’ answers; *n* (%*)			Importance of EOL care discussion *n* (%)		
			Important	Not important/DNK	*p*-value^c^
Family members or friends		614 (56)	492 (62)	122 (39)	*<*0.001
Healthcare providers		396 (36)	313 (40)	83 (27)	*<*0.001
Both family members and healthcare providers		328 (30)	271 (34)	57 (18)	*<*0.001
Needs^a^ and opportunities^b^ (%)					
Family members	Needs	75	84	53	*-*
	Opportunities	56	62	39	*-*
	Difference	19	22	14	*-*
Healthcare providers	Needs	80	86	64	*-*
	Opportunities	36	40	27	*-*
	Difference	44	46	37	*-*
Both family members and healthcare providers	Needs	65	74	40	*-*
	Opportunities	30	34	18	*-*
	Difference	35	40	21	*-*

*The percentages were calculated in proportion to the total number of participants (*n* = 1100). Multiple choices were allowed therefore the sum of the answers is not matching the number of participants.

^a^Needs = whose task is to talk with the patient about their EOL care wishes.

^b^Opportunities = there is someone with whom they could speak openly about death issues. DNK: Do not know.

^c^Fisher's Exact Test.

75% of participants thought that it was the task of the family members to talk with patients about their EOL care wishes, while 56% had a family member or friend with whom they could speak openly about death, dying and preparing for death. Regarding HCPs, eight out of ten participants believed that carrying out an EOL conversation was the HCPs’ task. However, less than four out of ten had someone among their HCPs with whom they could freely discuss death-related topics. A 35-point disparity was found between the majority who thought that both family members and HCPs should talk with the patients and the minority who had the opportunity for this discussion. Differences between needs and opportunities were even higher among those participants who considered the discussion of EOL care wishes important, especially concerning HCPs (46 points).

## Discussion

### Statement of principal findings

Our investigation revealed that conversations on EOL care were important for the general population. An almost equal level of engagement was expected from HCPs -particularly physicians- and family members in EOL conversations. Six out of ten participants believed that it was the GPs’ task to talk with the patients about their EOL care wishes. Compared to their needs, the general population had fewer opportunities to speak -especially with HCPs- about death, dying and preparing for death. Eight out of ten participants believed that carrying out EOL conversations was the HCPs’ task. However, less than four out of ten had someone among their HCPs with whom they could speak openly about this topic. The highest level of unmet communication needs was found among those who considered it important to discuss their EOL care wishes.

### Strengths and weaknesses of the study

Our study was the first survey in Hungary and -to our knowledge in a Central-European country- to explore the general population’s needs and opportunities to communicate their EOL care wishes. Another strength of the investigation was the large sample size, which was representative of the Hungarian adult population. Our study has some limitations. The questionnaire offered predetermined options and did not make it possible for participants to give different answers or explain their choices. The international comparison of our findings was challenging due to the different terminology and wording of the similar content questions.

### Findings in relation to other studies

Conversations regarding EOL care have become an important issue for the general population [1,2,6]. A national survey in Canada found that nine out of ten respondents believed discussing EOL preferences was important [4]. In the UK, eight out of ten adults thought that everyone should plan their EOL care [6], and in Norway, nine out of ten of the general public wished to participate in ACP and considered it advantageous for many patients [23]. In our study, seven out of ten Hungarians found EOL conversations important. This result was similar to international findings and indicated a considerable demand for discussing EOL care in Hungary.

Previous studies have confirmed that most people thought that EOL wishes should be primarily discussed with family members [2,7]. 93% of Canadians considered EOL discussions with family members or friends important, and 80% with HCPs [4]. In Norway, where -similarly to Hungary- ACP is seldom used, 69% of the general population thought HCPs should initiate ACP discussions, and 87% wanted to be accompanied by a family member [23]. Our results were different from these international findings. A lower but almost equal level of engagement was expected from family members and HCPs (75% and 80%) in EOL conversations. These results could be explained by the fact that talking about death and dying is still a challenge in Hungarian society [19,22] and people expect that HCPs start and facilitate open communication on EOL issues. In accordance with international studies [2,7], our study also revealed a high demand for physicians to initiate EOL conversations. Earlier studies have also highlighted that people preferred to discuss this topic with their attending physician rather than their GP [14,24]; however, the Norwegian general population most preferred GPs for ACP discussions [23]. GPs’ -presumed or perceived- limited competence and suboptimal collaboration with other specialists were common barriers identified by the literature [14,16]. These results were similar to our findings, and the underlying reasons might serve as explanations also for our outcomes. Nevertheless, six out of ten Hungarians thought that it was also the GPs’ task to talk with the patients about their EOL care wishes, and these results indicated that GPs -in line with international recommendations- would be in a good position to introduce ACP to patients in Hungary. The availability of well-trained GPs is essential for the broader application of ACP in primary care [15]. Specialized training programs and practice-oriented guidelines should be developed to support Hungarian GPs’ in implementation of ACP. Better collaboration between GPs and other specialists should also be encouraged. Collaborative models appear promising for effective ACP discussions [25,26,27], which is in accordance with Hungarians’ view that EOL conversation requires a cooperation between HCPs, and between HCPs and family members. A randomized controlled trial in Norway has confirmed that standardized education programs, clearly defined roles and responsibilities improve the implementation of ACP [28].

Countries differ regarding the proportion of the general population with the opportunity to speak freely about EOL care wishes. Eight out of ten British adults know who among their family members they could discuss their EOL plans with, and more than two-thirds of them could share their choices with their GP [6]. According to our analysis, less than six out of ten Hungarians had a family member or friend, and less than four out of ten had an HCP with whom they could speak openly about death, dying, and preparing for death. These opportunities for EOL conversations were lower compared to international results as well as compared to the needs of the Hungarian general population. The difference between needs and opportunities may be explained by the social denial of death and dying that is present among the general population and HCPs [18,19,22]. Perceptual bias may also be a reason: some people are comfortable talking about EOL issues, however, they believe others would be uncomfortable discussing them [6]. Regarding the HCPs, previous studies have revealed that many Hungarian HCPs felt unprepared to talk about EOL issues with patients [18,22]. The paternalistic approach -that still exists- in the Hungarian health care system may also hinder open communication between HCPs and patients about EOL issues and the shared decision-making on future care [19].

Training programs for HCPs -physicians, nurses, palliative care specialists- may increase the number of care providers prepared to participate in ACP discussions, resulting in more opportunities for people to discuss their EOL care wishes [12]. Public education is also a critical factor in ACP. Early ACP approaches focus on the population who will need it in the future [29]. Public awareness campaigns may facilitate open communication on death and dying, and inform the general public about the benefits of ACP and the potential role of GPs in its implementation [1,12,14,29]. Community-based ACP interventions (e.g. DöBra cards in Sweden [30]) may also benefit families who are uncomfortable talking about EOL issues. Evidence has shown that it was easier for physicians to initiate ACP discussions with informed patients and relatives [9]. The broader application of ACP could help HCPs better understand people’s needs regarding EOL care and it could be easier to have their wishes met at the end of life.

### Implications for practice, theory and policy

Understanding the needs and opportunities of the general population regarding EOL conversation may support the successful introduction of ACP in primary care in Hungary. Training programs for HCPs, particularly for GPs, may increase the number of HCPs available for ACP discussions. Research findings may help to identify priorities for public awareness campaigns. Our research adds valuable data to the topic of EOL conversation from the general population’s perspective, from the Central-European region. Further studies are needed to reveal similarities and differences between countries in terms of the general public’s wishes regarding EOL care and their needs and opportunities to discuss this topic to improve EOL planning.

## Ethics statement

The study followed the principles of the Declaration of Helsinki. Participants provided informed consent. Our research was a social study conducted among the general population; in accordance with the Hungarian legislation, the study did not require ethics committee approval.
